# Insulinotropic Effects of Neprilysin and/or Angiotensin Receptor Inhibition in Mice

**DOI:** 10.3389/fendo.2022.888867

**Published:** 2022-06-06

**Authors:** Nathalie Esser, Christine Schmidt, Breanne M. Barrow, Laura Cronic, Daryl J. Hackney, Stephen M. Mongovin, Meghan F. Hogan, Andrew T. Templin, Joseph J. Castillo, Rebecca L. Hull, Sakeneh Zraika

**Affiliations:** ^1^ Research Service, Veterans Affairs Puget Sound Health Care System, Seattle, WA, United States; ^2^ Division of Metabolism, Endocrinology & Nutrition, Department of Medicine, University of Washington, Seattle, WA, United States; ^3^ Laboratory of Immunometabolism and Nutrition, GIGA Infection, Immunity and Inflammation, University of Liège, Liège, Belgium

**Keywords:** angiotensin receptor-neprilysin inhibitor, insulin secretion, renin-angiotensin system, obesity, type 2 diabetes, sacubitril, valsartan, mouse

## Abstract

Treatment of heart failure with the angiotensin receptor-neprilysin inhibitor sacubitril/valsartan improved glycemic control in individuals with type 2 diabetes. The relative contribution of neprilysin inhibition versus angiotensin II receptor antagonism to this glycemic benefit remains unknown. Thus, we sought to determine the relative effects of the neprilysin inhibitor sacubitril versus the angiotensin II receptor blocker valsartan on beta-cell function and glucose homeostasis in a mouse model of reduced first-phase insulin secretion, and whether any beneficial effects are additive/synergistic when combined in sacubitril/valsartan. High fat-fed C57BL/6J mice treated with low-dose streptozotocin (or vehicle) were followed for eight weeks on high fat diet alone or supplemented with sacubitril, valsartan or sacubitril/valsartan. Body weight and fed glucose levels were assessed weekly. At the end of the treatment period, insulin release in response to intravenous glucose, insulin sensitivity, and beta-cell mass were determined. Sacubitril and valsartan, but not sacubitril/valsartan, lowered fasting and fed glucose levels and increased insulin release in diabetic mice. None of the drugs altered insulin sensitivity or beta-cell mass, but all reduced body weight gain. Effects of the drugs on insulin release were reproduced in angiotensin II-treated islets from lean C57BL/6J mice, suggesting the insulin response to each of the drugs is due to a direct effect on islets and mechanisms therein. In summary, sacubitril and valsartan each exert beneficial insulinotropic, glycemic and weight-reducing effects in obese and/or diabetic mice when administered alone; however, when combined, mechanisms within the islet contribute to their inability to enhance insulin release.

## Introduction

Sacubitril/valsartan is a first-in-class angiotensin receptor-neprilysin inhibitor that is increasingly being adopted for treatment of heart failure with reduced ejection fraction. It comprises moieties of the neprilysin inhibitor sacubitril and angiotensin II receptor blocker (ARB) valsartan at a 1:1 ratio in a crystalline complex, with the therapeutic goal of improving hemodynamic parameters through natriuretic peptide system enhancement with sacubitril and renin angiotensin system blockade with ARB (1). The use of sacubitril/valsartan has been associated with improved glycemic control (2, 3) and insulin sensitivity (4) in individuals with type 2 diabetes and/or obesity. Further, when compared to another renin angiotensin system inhibitor, namely the angiotensin-converting enzyme (ACE) inhibitor enalapril, sacubitril/valsartan resulted in greater reductions in HbA1c levels (2). The latter raises the possibility that the added benefit observed with sacubitril/valsartan could be a result of neprilysin inhibition.

Neprilysin is a ubiquitous peptidase that cleaves various peptides, some of which have glucoregulatory properties (5). It is found in both membrane-bound and soluble circulating forms (6), and we have shown that neprilysin is produced in pancreatic islets (7) where its inhibition/ablation enhances glucose-stimulated-insulin secretion (GSIS) (8, 9). Neprilysin protein and activity are upregulated in obesity and metabolic syndrome, and have been associated with insulin resistance, beta-cell dysfunction and impaired glucose tolerance (10, 11). Furthermore, we have reported that in the setting of a high fat diet, neprilysin deficient mice are protected against these deleterious effects (11). While there are no studies on the glucoregulatory effects of neprilysin inhibition alone in humans, reduction of neprilysin activity in preclinical studies has been associated with beneficial effects on glucose tolerance, insulin sensitivity and beta-cell function (9, 11–16).

Type 2 diabetes is associated with increased renin angiotensin system flux, which contributes to impaired beta-cell function and reduced insulin sensitivity (17). Clinical studies have reported that renin angiotensin system blockade with an ACE inhibitor or ARB is associated with a 25% reduction in the incidence of newly diagnosed diabetes in individuals with cardiovascular risk factors or congestive heart failure (18). Specifically, valsartan increased insulin secretion and sensitivity (19, 20) and reduced the risk of type 2 diabetes development in prediabetic individuals (21). Of note, neprilysin is also a component of the renin angiotensin system (22), though its role in cleaving angiotensin peptides is often overlooked.

Despite clinical data suggesting sacubitril/valsartan treatment is associated with better glycemic control in type 2 diabetes (2, 3), studies have not yet been performed to directly test whether it improves glucose tolerance and/or beta-cell function. Further, since prior studies have not included sacubitril and/or valsartan alone as comparators, the relative contribution of neprilysin inhibition versus angiotensin II receptor antagonism to the observed glycemic benefits remains unknown. Thus, in the present study, we evaluated the effect of sacubitril and valsartan, alone and combined in sacubitril/valsartan, on insulin secretion and glucose homeostasis in high fat-fed mice, including those with impaired insulin secretion.

## Materials And Methods

### Animal Models and Drug Treatments

Five-week old C57BL/6J male mice were purchased from the Jackson Laboratory (Bar Harbor, ME, USA). After an acclimation period of 5-12 days, a lateral saphenous vein blood sample was collected after a 16-hour fast to measure baseline fasting plasma glucose and insulin levels. Then, mice were fed a high fat diet (60% kcal fat, D12492; Research Diets Inc; New Brunswick, NJ, USA) for three weeks to induce weight gain and insulin resistance. Thereafter, mice were randomly assigned to receive once daily intraperitoneal injections on three consecutive days of either streptozotocin (STZ, 30 mg/kg; Sigma-Aldrich, St Louis, MO, USA) to induce diabetes, or vehicle (VEH, citrate buffered saline, pH 4.5) as control. This paradigm of multiple low dose STZ injections in high fat-fed mice is known to accelerate progression to beta-cell failure and recapitulate key features of human type 2 diabetes, namely loss of first-phase insulin release without a significant deficit in pancreatic insulin content (23, 24). After receiving STZ or VEH injections, mice were randomized to one of four treatment groups for eight weeks: high fat diet alone (control) or high fat diet supplemented with sacubitril (480 mg/kg diet, S080900, Toronto Research Chemicals Inc, Canada), valsartan (520 mg/kg diet, Novartis, Basel, Switzerland) or sacubitril/valsartan (1140 mg/kg diet, Novartis, Basel, Switzerland) with the goal of achieving a dose of 48, 52 and 114 mg/kg body weight per day, respectively. Sacubitril acts by binding neprilysin in the catalytic site, thereby inhibiting its activity *via* a competitive inhibition mode (25). The dose of sacubitril/valsartan was chosen in accordance with guidance from Novartis Pharmaceuticals Corporation, as well as a pilot study we performed in high fat-fed mice showing this dose was sufficient to significantly inhibit plasma neprilysin activity by 90%. Of note, 114 mg/kg/day of sacubitril/valsartan is equivalent to 100 mg/kg/day of its organic constituents sacubitril and valsartan, which excludes the mass of sodium and water contained within the product. Diet formulations were based on our prior experience and a pilot study in which each mouse consumed ~0.1 grams of food per gram body weight per day.

Mice were housed two per cage, with a 12 h light/12 h dark cycle, and had ad lib access to diets and water. Body weight, fed blood glucose levels, and food and water intake were assessed weekly. Daily drug intake was calculated based on food intake over the eight-week treatment period. The study was approved by the Institutional Animal Care and Use Committee of the VA Puget Sound Health Care System.

### Insulin and Glucose Tolerance Tests

At the end of the eight-week treatment period, intraperitoneal insulin tolerance tests (1 IU/kg; ITTs) were performed in conscious mice fasted for 3.5 hours, as previously described (11, 24). Two days later, intravenous glucose tolerance tests (1 g/kg; IVGTTs) were performed in pentobarbital (100 mg/kg) anesthetized mice fasted for 16 hours as previously described (16, 24), wherein blood was collected for measurement of glucose and insulin levels. Glucose elimination rate (Kg) during the IVGTT was computed as the negative slope of the natural log of glucose versus time from 10 to 45 minutes. First- and second-phase glucose-stimulated insulin secretion were assessed as the ratio of incremental areas under the curve (iAUC) for IVGTT insulin over glucose for 0-5 minutes and 5-45 minutes, respectively. Total insulin secretion was assessed as the iAUC for IVGTT insulin from 0 to 45 minutes.

### Tissue Collection, Pancreatic Insulin and Glucagon Content and Histological Assessments

Following completion of the IVGTTs, mice were euthanized and epididymal fat, inguinal fat and brown adipose tissue mass were recorded. A small portion of the pancreas (tail) was weighed and homogenized in acid-ethanol for evaluation of insulin and glucagon contents (expressed per total protein content). The same pancreatic region from each mouse was extracted in order to avoid variability that may arise due to random sampling. The remaining pancreas was excised, weighed and fixed in 10% neutral-buffered formalin overnight, paraffin-embedded and sectioned at 4 μm thickness. Two sections (100 μm apart) per mouse were deparaffinized and stained with anti-insulin (1:2000; clone K36AC10, Sigma-Aldrich, St Louis, MO, USA) and anti-glucagon (1:2000; EP3070, Abcam, Cambridge, MA, USA) antibodies followed by Alexa Fluor 488-conjugated anti-mouse and Alexa Fluor 546-conjugated anti-rabbit to visualize islet beta- and alpha cells, respectively. The sections were then counterstained with Hoechst 33258 (2 μg/ml) to visualize cell nuclei. Morphometric analyses were performed using NIS-Elements AR software on a Nikon Ni-E microscope system (Nikon USA, Melville, NY, USA). The data collector was blinded to the identity of each specimen. Histological assessments were made for all islets visible on each section, averaging 23 ± 9 islets per mouse. Section area, islet area, insulin and glucagon areas were quantified and beta-cell and alpha-cell area were expressed relative to islet area (Σ insulin area/Σ islet area × 100). Beta-cell and alpha-cell mass were then calculated as following: beta-cell or alpha-cell area/pancreas area × total pancreas weight, as previously reported (26–30).

### Glucose, Insulin, Glucagon, Glycerol and Free Fatty Acids Assays

For baseline bleeds and IVGTTs, plasma glucose levels were determined using the glucose oxidase method. For weekly measurements and ITTs, blood glucose levels were measured using an Accu-Chek Aviva Plus glucometer (Roche, Basel, Switzerland). Insulin levels in plasma and pancreas extracts were determined using a Mouse Ultrasensitive Insulin ELISA (Alpco, Salem, NH, USA). Glucagon levels in pancreas extracts were determined using Mercodia’s Glucagon ELISA (Mercodia, Uppsala, Sweden). For free fatty acids and glycerol levels, lateral saphenous vein blood was collected at the end of the eight-week treatment period from 16-hour fasted mice, and were measured using a Free Fatty Acid Quantification Assay kit (Abcam, Cambridge, MA, USA) and Free Glycerol Determination Assay (Sigma-Aldrich, St Louis, MO, USA), respectively.

### Neprilysin Activity Assay

Lateral saphenous vein blood from conscious, non-fasted mice was collected following eight weeks of treatment. Plasma neprilysin activity was assessed by a fluorometric enzyme method, as previously described (11). Each plasma sample was assayed in both the absence and presence of a specific neprilysin inhibitor (DL-thiorphan, Sigma-Aldrich, St Louis, MO, USA) to differentiate neprilysin activity from non-specific endopeptidase activity.

### *In Vitro* Assessment of Insulin Secretion

Islets from 10-12 week old lean C57BL/6J male and female mice were isolated by collagenase digestion and recovered overnight in RPMI 1640 media. Islets were then pre-incubated for 90 minutes in Krebs buffer containing 2.8 mmol/l glucose, with valsartan (1 μmol/l; Sigma-Aldrich, St Louis, MO, USA) being added during the last 15 minutes. Triplicate batches of islets were then transferred to Krebs buffer containing 2.8 or 20 mmol/l glucose alone, or plus 100 nmol/l angiotensin II (AnaSpec Inc, Freemont, CA, USA), 1 μmol/l valsartan and/or 1 μmol/l sacubitrilat (Sigma-Aldrich, St. Louis, MO, USA). After 60 minutes, supernatant was collected for insulin assay (Alpco, Salem, NH, USA). Doses of angiotensin II and valsartan were based on prior literature (31, 32). The dose of sacubitrilat, the active metabolite of sacubitril, was chosen to achieve a 1:1 molar ratio with valsartan, as administered in the mouse diet.

### Statistical Analyses

Data are presented as mean ± SEM. All outliers were included in the figures and data analysis. Time-course data were analyzed *via* two-way analysis of variance (ANOVA) with time and drugs being the two variables. Mean data were compared among treatment groups by one-way ANOVA. Dunnet correction for multiple comparisons was used for post-hoc analyses. A Student t-test was used when two groups were compared (eight-week treatment vs. baseline, STZ vs. VEH within the same treatment group). A p<0.05 was considered statistically significant. All statistical analysis was performed using Prism 8 (GraphPad Software, San Diego, CA, USA).

## Results

### Plasma Neprilysin Activity and Drug Intake Amongst Treatment Groups

**Figure 1:**
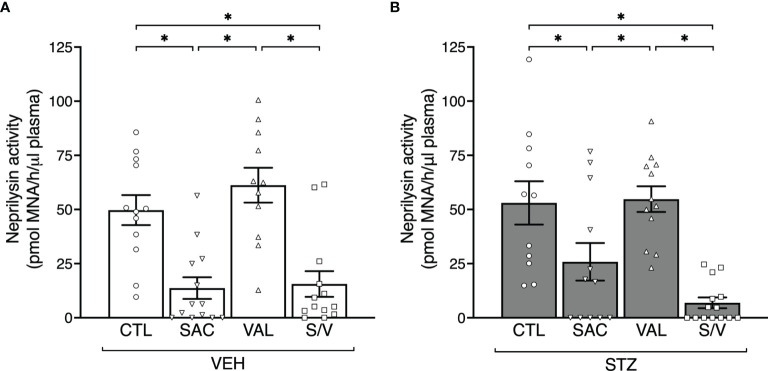
Sacubitril and sacubitril/valsartan but not valsartan reduce plasma neprilysin activity. Plasma neprilysin activity after 8 weeks of treatment with control (CTL), sacubitril (SAC), valsartan (VAL) or sacubitril/valsartan (S/V) in high fat-fed mice that received vehicle (**A**; VEH) or streptozotocin (**B**; STZ) injections. n=11-14/group. *p<0.05.

At the end of the eight-week treatment period, in VEH-injected mice, plasma neprilysin activity was reduced by 68.7 ± 11.9% and 72.5 ± 10.0% in sacubitril- and sacubitril/valsartan-treated mice respectively, compared to mice receiving the control diet (**Figure 1A**). Similarly, STZ-injected mice treated with sacubitril or sacubitril/valsartan displayed decreased plasma neprilysin activity by 87.0 ± 4.7% and 51.3 ± 16.3% respectively, compared to control (**Figure 1B**). In both VEH and STZ groups, valsartan did not affect plasma neprilysin activity compared to control (**Figures 1A, B**). Amongst the mice that received VEH injections, food and water intake over the pre- and post-treatment period did not differ between any of the groups (**Supplementary Figures 1A, C**). In contrast, in mice that received STZ injections, those treated with sacubitril consumed significantly less food and water than controls or those treated with sacubitril/valsartan (**Supplementary Figures 1B, D**). The average drug intake over the eight-week treatment period was similar amongst all groups of mice, but approximately 25-30% less than the target doses for all treatment groups (**Supplementary Figure 1E**).

### Sacubitril and Valsartan but not Sacubitril/Valsartan Lower Glucose Levels in STZ Mice

**Figure 2:**
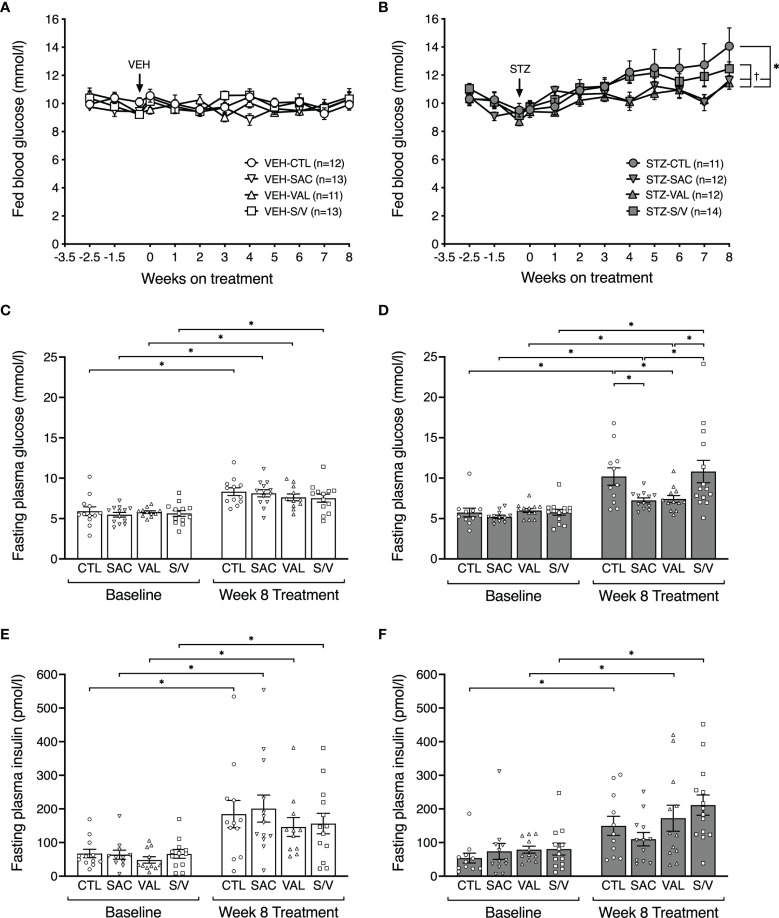
Sacubitril and valsartan but not sacubitril/valsartan lower fed and fasting glucose in diabetic mice. Fed blood glucose levels over time in vehicle (**A**;VEH) and streptozotocin (**B**; STZ)- injected high fat-fed mice treated for 8 weeks with control (circles, CTL), sacubitril (inverted triangles, SAC), valsartan (triangles, VAL) or sacubitril/valsartan (squares, S/V). The arrow shows when VEH or STZ was injected. n=11-14/group. *p<0.05 SAC and VAL vs CTL, †p<0.05 SAC and VAL vs S/V. 16-hour fasted plasma glucose **(C, D)** and insulin **(E, F)** measured in VEH **(C, E)** and STZ **(D, F)** groups of mice at baseline (week -3.5) and at the end of the 8-week treatment period with CTL, SAC, VAL or S/V. n=11-14/group. *p<0.05.

Fed and fasting glucose levels did not differ at baseline (week -3.5) amongst groups of mice (**Figures 2A–D**). Throughout the study period, fed glucose levels remained comparable in all groups of VEH-injected mice and did not significantly increase compared with their baseline levels (**Figure 2A**). Similarly, fasting glucose levels at eight weeks post-treatment were similar amongst the groups of VEH-injected mice, although significantly increased compared to their baseline levels (**Figure 2C**). As expected, STZ increased fed and fasting glucose in control mice (**Figures 2B, D**; p<0.05 for both vs. VEH control mice). In STZ-injected mice, sacubitril and valsartan lowered fed glucose over time (**Figure 2B**) and fasting glucose levels at eight weeks post-treatment (**Figure 2D**), while sacubitril/valsartan had no effect (**Figures 2B, D**). Further, fed and fasting glucose levels were significantly lower in both valsartan and sacubitril-treated STZ-injected mice when compared with sacubitril/valsartan-treated STZ-injected mice (**Figure 2B**).

While an increase in fasting plasma insulin levels from baseline to week eight was observed in all groups except STZ-injected mice treated with sacubitril, these were not significantly different among the four treatment groups within both VEH- (**Figure 2E**) and STZ- (**Figure 2F**) injected mice.

### Sacubitril and Valsartan but not Sacubitril/Valsartan Protect Against STZ-Induced Defects in Insulin Secretion

**Figure 3:**
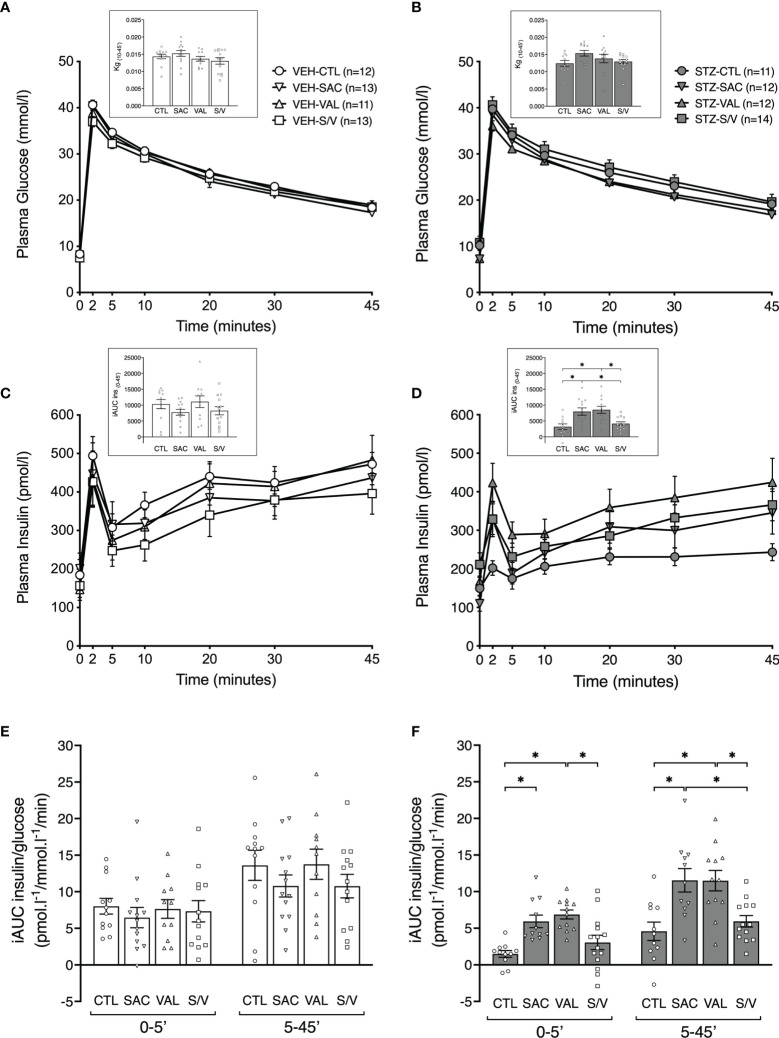
Sacubitril and valsartan but not sacubitril/valsartan increase glucose-stimulated insulin secretion in diabetic mice. Plasma glucose **(A, B)** and insulin **(C, D)** during an IVGTT in vehicle [**(A, C)** VEH]- and streptozotocin [**(B, D)** STZ]-injected high fat-fed mice treated for 8 weeks with control (circles; CTL), sacubitril (inverted triangles; SAC), valsartan (triangles; VAL) or sacubitril/valsartan (squares; S/V). The insets in A and B represent the rate of glucose disappearance (Kg) computed from 10 to 45 minutes during the IVGTT. The insets in C and D represent the incremental areas under the curve (iAUC) for insulin from 0 to 45 minutes. First-phase [0-5’] and second-phase [5-45’] glucose-stimulated insulin secretion **(E, F)** during the IVGTT in VEH **(E)**- and STZ **(F)**- injected mice treated for 8 weeks with CTL, SAC, VAL or S/V. n=11-14/group. *p<0.05.

To determine whether the treatments were associated with enhanced insulin release, IVGTTs were performed at the end of the eight-week treatment period. Plasma glucose levels are shown in **Figures 3A, B**. STZ-injected control mice tended to exhibit a reduced rate of glucose disappearance (Kg) compared to VEH-injected control mice (**Figures 3A, B insets;** p=0.089), suggesting their glucose tolerance may be impaired. None of the treatments significantly altered Kg in either STZ- or VEH-injected mice (**Figures 3A, B insets**). As expected, STZ-injected control mice displayed a marked reduction in insulin secretion over the 45 minutes of the IVGTT, compared to VEH-injected control mice (**Figures 3C, D and insets;** p=0.001), with a significant decrease in both first- and second-phase GSIS (**Figures 3E, F**, p<0.05 vs. VEH control mice). First- and second-phase GSIS were similar amongst treatment groups within VEH-injected mice (**Figure 3E**). In STZ-injected mice, sacubitril and valsartan markedly and similarly enhanced first- and second-phase GSIS, while sacubitril/valsartan did not (**Figure 3F**).

### Sacubitril, Valsartan and Sacubitril/Valsartan do not Alter Beta-Cell and Alpha-Cell Mass

**Figure 4:**
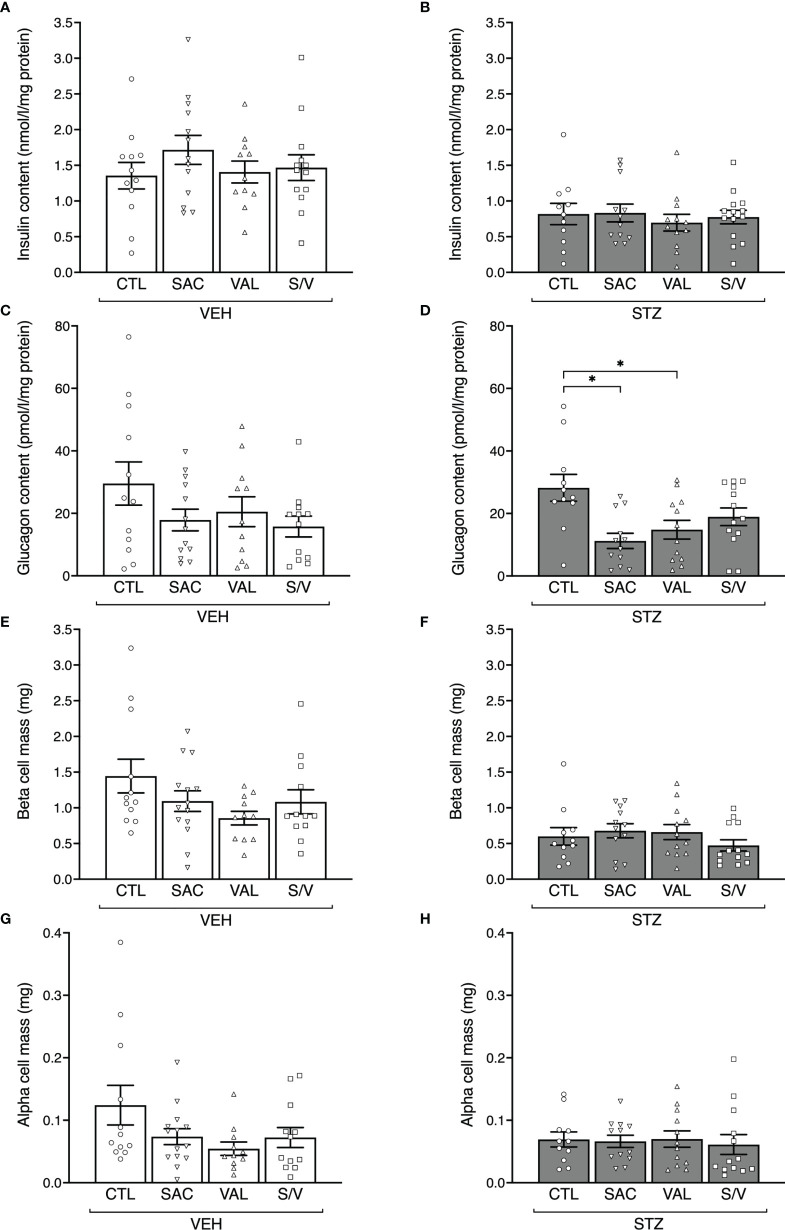
Sacubitril, valsartan and sacubitril/valsartan do not affect beta- and alpha-cell mass. Insulin content **(A, B)**, glucagon content **(C, D)**, beta-cell mass **(E, F)** and alpha-cell mass **(G, H)** at the end of the 8-week treatment period in vehicle (**A, C, E, G**; VEH) and streptozotocin (**B, D, F, H; ** STZ) injected high fat-fed mice treated with control (CTL), sacubitril (SAC), valsartan (VAL) or sacubitril/valsartan (S/V). n=11-14/group. *p<0.05.

To evaluate whether changes in insulin release could be explained by changes in insulin content or beta-cell mass, these parameters were assessed at the end of the eight-week treatment period, at which time glucagon content and alpha-cell mass were also determined. Pancreas weight did not differ amongst any groups of mice (**Table 1**). STZ injection in control mice was associated with a significant decrease in insulin content and beta-cell mass (**Figures 4A, B, E, F**; p<0.05 for both vs. VEH control mice), while glucagon content and alpha-cell mass were unaltered (**Figures 4C, D, G, H**; p>0.05 for both vs. VEH control mice). Within both VEH and STZ-injected groups of mice, insulin content did not differ among the four treatment groups (**Figures 4A, B**). Similarly, in VEH-injected mice, glucagon content did not differ among the four treatment groups (**Figure 4C**). In STZ-injected mice, glucagon content was decreased with sacubitril or valsartan, but not sacubitril/valsartan treatment when compared with untreated mice (**Figure 4D**). However, glucagon content did not differ among STZ-injected mice treated with sacubitril, valsartan or sacubitril/valsartan (**Figure 4D**). Beta- and alpha-cell mass were similar among the four treatment groups in both VEH- and STZ-injected mice (**Figures 4E–H**).

**Table 1:** Pancreas and fat pad mass in high fat-fed mice that received vehicle (VEH) or streptozotocin (STZ) injections and were treated for 8 weeks with control (CTL), sacubitril (SAC), valsartan (VAL) or sacubitril/valsartan (S/V).

	VEH				STZ			
	CTL (n=12)	SAC (n=13)	VAL (n=11)	S/V (n=13)	CTL (n=11)	SAC (n=12)	VAL (n=12)	S/V (n=14)
Pancreas (g)	0.332 ± 0.03	0.301 ± 0.03	0.298 ± 0.01	0.311 ± 0.02	0.299 ± 0.02	0.296 ± 0.01	0.319 ± 0.01	0.299 ± 0.02
Epididymal fat mass (g)	2.75 ± 0.21	2.43 ± 0.27	2.28 ± 0.20	2.10 ± 0.19	2.41 ± 0.17	2.31 ± 0.16	2.19 ± 0.23	2.10 ± 0.18
Inguinal fat mass (g)	1.60 ± 0.18	1.51 ± 0.19	1.32 ± 0.15	1.10 ± 0.15	1.35 ± 0.15	1.35 ± 0.15	1.08 ± 0.17	1.11 ± 0.12
Brown adipose tissue mass (mg)	171 ± 12	155 ± 11	124 ± 8*	125 ± 10*	127 ± 9*	124 ± 10	136 ± 16	151 ± 15

*p<0.05 vs VEH-CTL.

### Sacubitril, Valsartan and Sacubitril/Valsartan do not Alter Insulin Sensitivity

**Figure 5:**
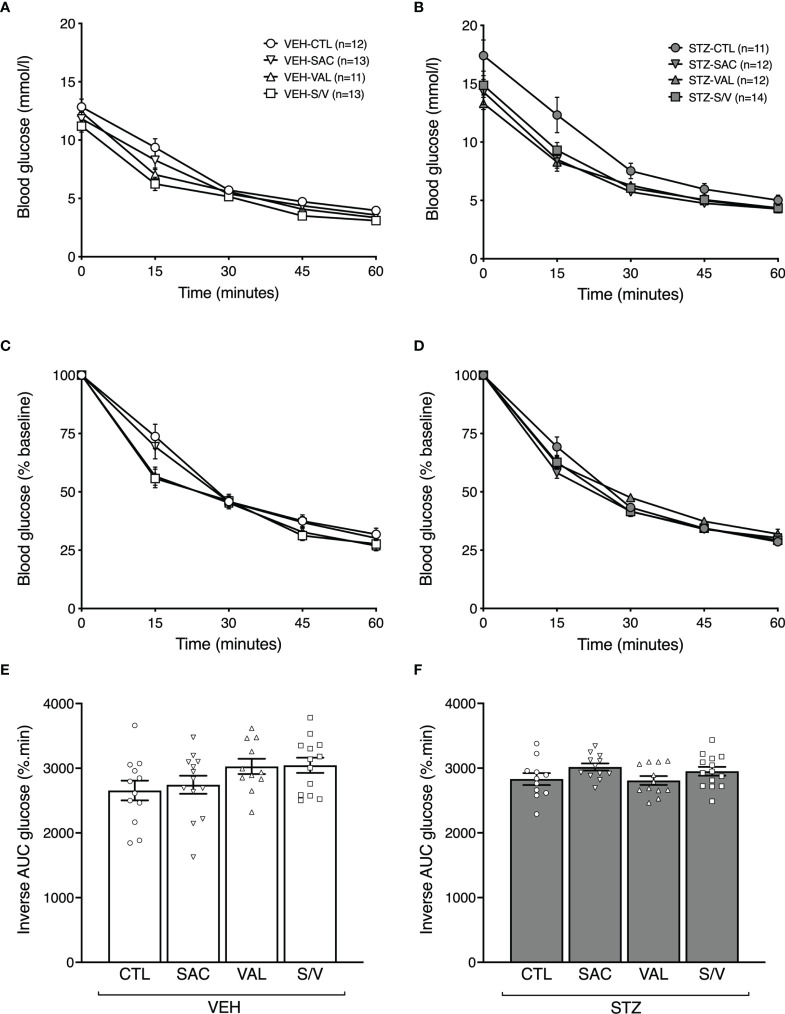
Sacubitril, valsartan and sacubitril/valsartan do not alter insulin sensitivity in both obese and diabetic mice. Blood glucose levels, as absolute values **(A, B)** and as % of baseline **(C, D)**, during an intraperitoneal insulin tolerance test in high fat-fed mice injected with vehicle (**A, C**; VEH) or streptozotocin (**B, D**; STZ) and treated for 8 weeks with control (circles, CTL), sacubitril (inverted triangles, SAC), valsartan (triangles, VAL) or sacubitril/valsartan (squares, S/V). Inverse area under the % of baseline glucose curve accounting for adjustment for glucose baseline in VEH **(E)**- or STZ **(F)**-injected mice treated for 8 weeks with CTL, SAC, VAL or S/V. n=11-14/group.

To determine whether effects of the drugs to improve GSIS were accompanied by enhanced insulin sensitivity, ITTs were performed at the end of the eight-week treatment period. Absolute blood glucose levels during the ITT for VEH- and STZ-injected mice are shown in **Figures 5A, B**, respectively. When accounting for differing baseline glucose levels amongst the groups of mice (**Figures 5C, D**) or when computed as inverse area under the curve (**Figures 5E, F**), no significant differences in insulin sensitivity were observed amongst any of the treatment groups in either VEH- or STZ-injected mice.

### Effect of the Drug Treatments on Body Weight, Fat Pad Mass and Lipolysis

**Figure 6:**
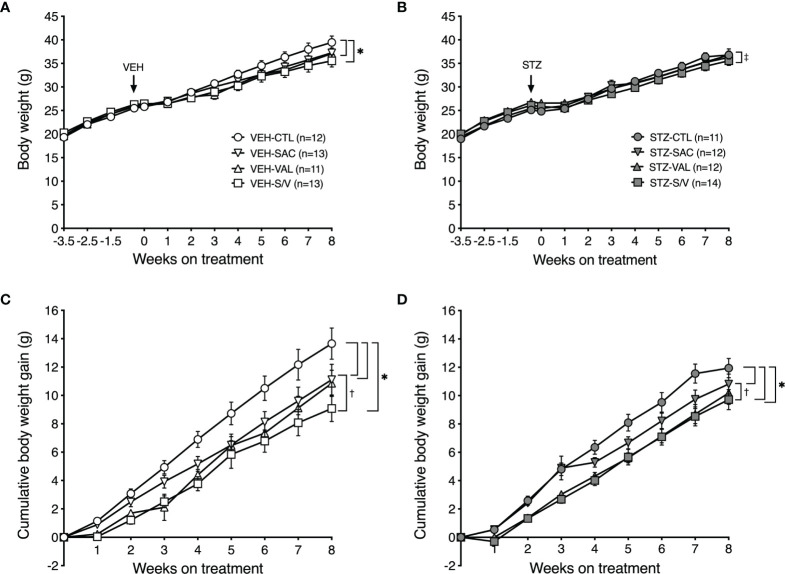
Sacubitril, valsartan and sacubitril/valsartan reduce body weight gain in both obese and diabetic mice. Body weight over time in high fat-fed mice injected with vehicle (**A**; VEH) or streptozotocin (**B**; STZ), and then treated for 8 weeks with control (circles, CTL), sacubitril (inverted triangles, SAC), valsartan (triangles, VAL) or sacubitril/valsartan (squares, S/V). The arrow shows when VEH or STZ was injected. Cumulative body weight gain over the 8-week treatment period in high fat-fed mice injected with VEH **(C)** or STZ **(D)** and treated with CTL, SAC, VAL or S/V. n=11-14/group. *p<0.05 SAC, VAL and S/V vs. CTL, ^‡^p<0.05 VAL vs. S/V, ^†^p<0.05 SAC vs. S/V.

At baseline (week -3.5), body weight did not differ amongst the groups of mice (**Figures 6A, B**). As expected with the high fat diet, body weight significantly increased throughout the study period in all groups of mice (p<0.0001 for each group). Over the pre-treatment period, the increase in body weight was comparable in mice that went on to receive either VEH (**Figure 6A**) or STZ (**Figure 6B**) injections. Within the group of mice that received VEH injections, all drugs decreased body weight gain (**Figure 6C**), with sacubitril/valsartan being more effective than sacubitril (**Figure 6C**). A similar effect of the drug treatments on body weight was observed within the group of mice that received STZ injections (**Figure 6D**).

To determine whether the effect of the drugs to attenuate body weight gain was associated with decreased fat deposition, fat pad mass was assessed at the end of the eight-week treatment period. There were no significant differences in epididymal or inguinal fat pad mass amongst treatment groups in either VEH- or STZ-injected mice (**Table 1**). Brown adipose tissue mass was significantly lower in valsartan- and sacubitril/valsartan-treated mice injected with VEH but did not differ amongst the treatment groups in STZ mice (**Table 1**).

Further, given that increased natriuretic peptide levels due to neprilysin inhibition and renin angiotensin system blockade could enhance lipid mobilization (33, 34), fasting plasma glycerol and free fatty acids levels, two lipolysis markers, were measured at the end of the eight-week treatment period. Both glycerol (**Supplementary Figures 2A, B**) and free fatty acids (**Supplementary Figures 2C, D**) levels were similar amongst the treatment groups within VEH- and STZ-injected mice.

### Islet-Specific Effects of Sacubitril, Valsartan and Sacubitril/Valsartan to Protect Against Angiotensin II-Induced Defects in Insulin Secretion

**Figure 7:**
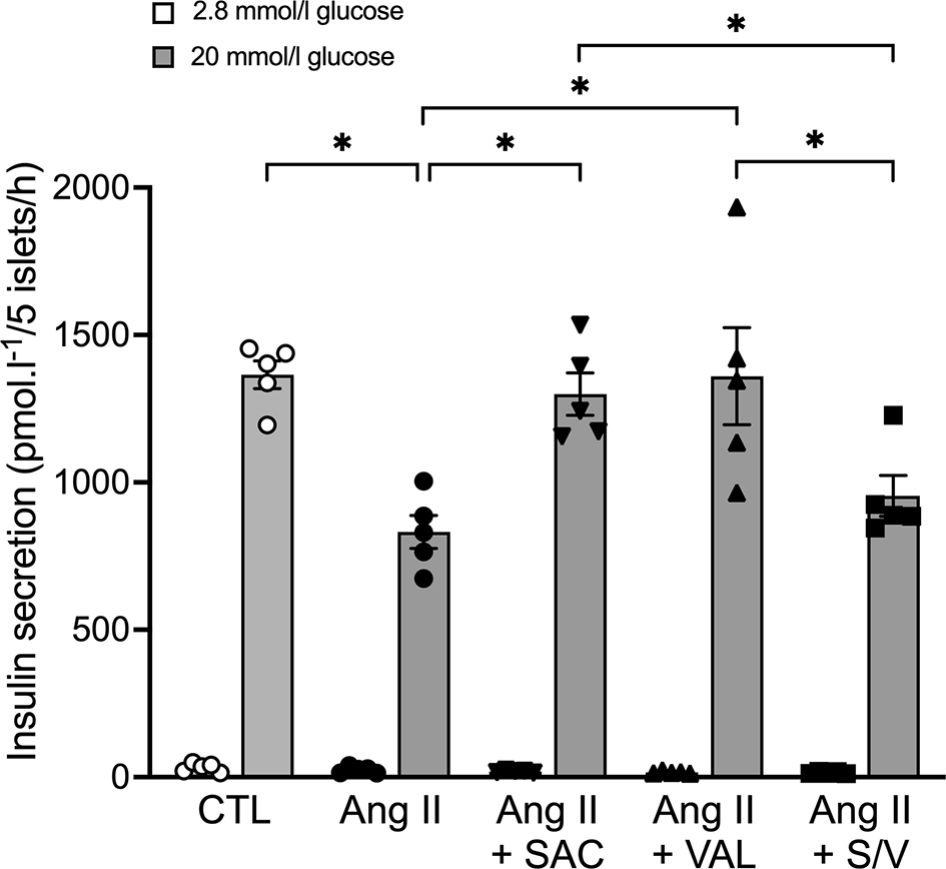
Sacubitrilat and valsartan alone but not combined, increase glucose-stimulated insulin secretion in angiotensin II-treated islets. Insulin secretion in response to 2.8 mmol/l (white bars) and 20 mmol/l (grey bars) glucose in isolated islets from lean C57BL6/J mice following incubation with vehicle (CTL, open circles), 100 nmol/l angiotensin II alone (Ang II, black circles), 100 nmol/l angiotensin II + 1 μmol/l sacubitrilat (SAC, black inverted triangles), 1 μmol/l valsartan (VAL, black triangles), or 1 μmol/l sacubitrilat and 1 μmol/l valsartan (S/V, black squares). Data are mean ± S.E.M of 5 independent experiments. *p<0.05.

Given that neprilysin inhibition (9, 16) or renin angiotensin system blockade (31, 32) both enhance insulin secretion in isolated islets, we sought to determine whether an interaction between sacubitril and valsartan at the level of the islet renin angiotensin system could explain the inability of sacubitril/valsartan to elicit an insulinotropic effect. To do this, an *in vitro* model of angiotensin II-induced beta-cell dysfunction in which ARBs have previously been shown to be protective (31, 32) was utilized. As expected, angiotensin II treatment reduced GSIS, compared to control (**Figure 7**). Both sacubitril alone and valsartan alone enhanced GSIS in angiotensin II-treated islets; however, sacubitril/valsartan had no effect (**Figure 7**). Basal insulin secretion did not change with any of the treatments.

## Discussion

We evaluated for the first time the potential benefits of sacubitril/valsartan versus sacubitril or valsartan on beta-cell function and glucose homeostasis in obese and/or diabetic mice. We found sacubitril and valsartan, but surprisingly not their combination, improves insulin secretory dysfunction and reduces hyperglycemia in STZ-treated mice.

In line with our previous study (16), we found pharmacological neprilysin inhibition with sacubitril exerts beneficial insulinotropic and glycemic effects in mice with beta-cell dysfunction. We recently proposed neprilysin inhibition could improve beta-cell function by enhancing signaling from intra-islet glucagon-like peptide-1 (GLP-1). Indeed, this insulinotropic peptide is a substrate of neprilysin (35, 36), and its circulating levels increased postprandially following neprilysin inhibition in humans (37). In our previous study, pharmacological neprilysin inhibition did not increase circulating active GLP-1 levels in a mouse model of beta-cell dysfunction, but it improved intravenous glucose-stimulated insulin secretion *in vivo* in a GLP-1 receptor-dependent manner (16). Further, acute inhibition of islet neprilysin *in vitro* also enhanced glucose-stimulated insulin secretion in a GLP-1 receptor-dependent manner (9, 16). Thus, preservation of intra-islet GLP-1 may be a mechanism by which sacubitril exerts its beneficial effects on beta-cell function in the current study. Since neprilysin has broad substrate specificity, it is also possible that levels of other substrates known to enhance beta-cell function, such as gastric inhibitory polypeptide (35), peptide YY (38), cholecystokinin-8 (CCK-8) (39) or gastrin (40), were elevated following sacubitril treatment.

Obesity and type 2 diabetes are associated with a pathological activation of the renin angiotensin system. Clinical studies have shown renin angiotensin system blockade with valsartan prevents new-onset diabetes, and improves beta-cell function and insulin sensitivity in individuals at risk of developing type 2 diabetes (20, 21). In agreement with others (41), we found valsartan lowers glucose levels and improves beta-cell function in our mouse model of reduced first-phase insulin secretion. Although the precise mechanisms by which ARBs exert beneficial effects on glucose metabolism are not fully understood, several hypotheses have been suggested. These include an ability to lower inflammatory markers in adipose tissue and plasma, suggesting beneficial effects on beta-cell function may be secondary to reduced systemic inflammation (41). At the level of the islet, blood flow is increased (42, 43) and this is associated with enhanced insulin secretion (43). Similarly, inhibition of angiotensin II type 1 receptor (AT1R) in islets upregulates GLUT-2 and glucokinase, enhances (pro)-insulin biosynthesis, and increases levels of insulin/PI3K-AKT signaling proteins, all effects that were shown to correlate with improved insulin secretion (44). It has also been suggested that ARBs activate the alternative renin angiotensin system, namely ACE2/angiotensin- (1–7)/Mas receptor axis (45, 46), which counteracts effects of the canonical ACE/angiotensin II/AT1R axis, thereby improving insulin sensitivity and secretion (47). A local renin angiotensin system has been described in islets (31, 42, 48), wherein upregulation of its canonical axis under metabolic stress results in beta-cell dysfunction and death (32, 48–51) that can be largely attenuated by ARBs (31, 32, 48). In our study, valsartan did not affect pancreatic insulin content or beta-cell mass; it is therefore unlikely that the improvement in beta-cell function was explained by changes in insulin biosynthesis and beta-cell survival. Similarly, alpha-cell mass was unchanged, whereas pancreatic glucagon content was reduced. The latter may support improved glycemia, though additional studies are needed to ascertain if this is indeed the case. Notwithstanding, based on our *in vitro* study, we do feel that the local islet renin angiotensin system may play a critical role in the effect of valsartan to enhance beta-cell function.

Given the beneficial insulinotropic and glycemic effects observed with sacubitril and valsartan alone, we expected that when combined they would have additive and/or synergistic effects. Surprisingly, we found no such effect on insulin secretion or glucose levels in diabetic mice. It is therefore possible that combining a neprilysin inhibitor with an ARB elicits competing effects, which abolish the benefits of either alone. This drug interaction is difficult to examine, as simply blocking or activating any given candidate pathway would result in bypassing the potential interaction itself. Thus, while our study lacks mechanistic insight into the competing effects of sacubitril versus valsartan, the literature and our previous studies provide some clues. First, it is possible an interaction between the two drugs occurs at the level of GLP-1 metabolism. Indeed, neprilysin inhibition limits GLP-1 degradation (35–37) and potentiates GSIS *via* intra-islet GLP-1 receptor signaling (9, 16). However, such effects may be diminished in the presence of ARBs – this is because ARBs can limit GLP-1 secretion. For example, it has been reported that angiotensin II stimulates GLP-1 secretion from primary cultures of mouse and human colon, but that this effect is antagonized by the ARB candesartan (52). Second, since neprilysin is a component of the renin angiotensin system (22), an interaction between sacubitril and valsartan might involve this system. Specifically, both neprilysin inhibition (53) and AT1R blockade (46, 54) increase levels of angiotensin-(1-7), which exerts beneficial glycemic effects (55). However, neprilysin inhibition also prevents production of angiotensin-(1-2), the insulinotropic dipeptide that we have shown is responsible for angiotensin-(1-7)’s insulinotropic properties (56). Thus, sacubitril and valsartan may be modulating angiotensin-(1-7)/angiotensin-(1-2) levels and subsequent downstream signaling *via* mechanisms that compete when the drugs are combined. Finally, another component of the renin angiotensin system, namely ACE, may be playing a role in the observed effects of sacubitril versus valsartan. That is, it is possible valsartan increases ACE activity in our diabetic mice, as previously reported in plasma from humans treated with valsartan for one week (57). This would reduce levels of glucoregulatory/insulinotropic peptides that are cleaved by ACE, such as PYY, CCK-8 and gastrin (58–60). Some of these peptides are also cleaved by neprilysin (38–40). Thus, sacubitril could have opposing effects to valsartan on levels of substrates that are common to ACE and neprilysin. This may only occur in a chronic treatment setting since studies in healthy humans show that some of these substrates are increased postprandially following acute sacubitril/valsartan administration (61). Further work is required to unravel the potential complex interactions that may explain the effects of sacubitril and valsartan in our model. As a starting point, we assessed whether such competing effects occur at the level of the islet by studying isolated islets treated with angiotensin II and sacubitril/valsartan. This *in vitro* model is relevant to our *in vivo* study because increased systemic and pancreatic islet renin angiotensin system observed in diabetic mice can impair insulin secretion (17, 31, 32, 49–51) *via* a direct effect on the islet. In isolated islets treated with angiotensin II, we found that sacubitril alone or valsartan alone, but not their combination, could enhance insulin secretion. While additional studies are needed to understand the molecular mechanisms underlying these findings, our data clearly identify the islet as a site for potentially competing effects of sacubitril versus valsartan.

Of note, our findings with sacubitril/valsartan contrast with some reports from clinical studies showing improved glycemic control in both diabetic and non-diabetic patients with heart failure (2, 3). It is possible that improvement in severity of heart failure itself with sacubitril/valsartan may have contributed to the better glycemic control observed in these individuals. Indeed, while it has long been recognized that type 2 diabetes is a risk factor for heart failure (62, 63), heart failure has also been associated with increased risk of developing type 2 diabetes (64). In keeping with this, restoration of normal cardiac output with implantation of left ventricular assist devices improves glycemic control in patients with congestive heart failure (65). Further, heart failure is associated with insulin resistance (66) and increased sympathetic nerve activity (67), the latter known to inhibit insulin secretion and stimulate both hepatic gluconeogenesis and glycogenolysis (68). Interestingly, treatment of obese hypertensive patients with sacubitril/valsartan for 8 weeks was associated with decreased levels of epinephrine and improved insulin sensitivity in one study (4), but no effect on fasting glucose levels in a more recent study (69). To better clarify the role of heart failure in the glycemic effects of sacubitril/valsartan and its components, further studies in a mouse model that recapitulates the clinical features of heart failure in the setting of diabetes would be valuable. Conversely, clinical studies with sacubitril/valsartan in individuals without heart failure would shed light on whether factors associated with heart failure determine the glucose-lowering effect of the drug. Indeed, a study in which humans without heart failure were acutely administered sacubitril/valsartan showed that glucose and insulin levels were unchanged (37). This supports the idea that sacubitril/valsartan may only confer glycemic benefit in the setting of heart failure.

In contrast to the absence of glycemic and insulinotropic effects with sacubitril/valsartan in diabetic mice, we found sacubitril/valsartan reduced body weight gain in obese and/or diabetic mice. This suggests mechanisms other than those regulating glucose homeostasis may be involved in body weight control, and do not result in competing effects when sacubitril and valsartan are combined. For example, natriuretic peptides, whose plasma levels are increased with neprilysin inhibition, could protect against weight gain by improving lipid mobilization and oxidation (34). Previous studies have reported that ARB treatment is associated with reduced body weight gain and suggested that potential mechanisms underlying this effect on fat metabolism could involve decreased adipogenesis and fat accumulation, increased lipolysis and adiponectin, and promotion of energy expenditure (33, 70, 71). In our study, the effects on body weight did not seem to be explained by differences in food intake. Further, none of the treatments were associated with changes in plasma glycerol and free fatty acids levels, suggesting lipolysis was not increased. Finally, it cannot be excluded that changes in energy expenditure or body fluid balance underlie the effects on body weight gain.

In understanding our findings with sacubitril/valsartan, we also need to consider that mice consumed ~25-30% less food than expected during the treatment period, and so they received only ~70-75% of the target drug dosage. That said, plasma neprilysin activity following either sacubitril/valsartan or sacubitril treatment was significantly reduced, and to the same extent in both groups. Thus, it seems unlikely that a lack of maximal neprilysin inhibition by sacubitril/valsartan could explain the discrepant results between sacubitril/valsartan and sacubitril treatment. Importantly, our results confirmed the importance of including a neprilysin inhibitor arm for interpretation of the data. Without this critical arm of the study, one interpretation of the data comparing ARB and ARB plus neprilysin inhibition would be that reducing neprilysin action elicits deleterious glycemic and insulinotropic effects.

In conclusion, neprilysin inhibition with sacubitril alone and angiotensin II receptor antagonism with valsartan alone exerted beneficial insulinotropic and glycemic effects in diabetic mice only. Surprisingly, these effects were not observed when the drugs were combined in sacubitril/valsartan, which may be explained, in part, by competing effects at the level of the islet. It is possible that other tissues are similarly subject to such competing effects. Thus, additional studies are needed to understand the molecular mechanisms underlying these outcomes and also whether sacubitril/valsartan is only effective in improving glycemic control in the setting of heart failure.

## Data Availability Statement

The original contributions presented in the study are included in the article/**Supplementary Material**. Further inquiries can be directed to the corresponding author.

## Ethics Statement

The animal study was reviewed and approved by the Institutional Animal Care and Use Committee of the VA Puget Sound Health Care System.

## Author Contributions

NE designed the study, performed experiments, analyzed and interpreted data, and wrote the manuscript. CS, BB, DH and LC performed experiments, analyzed and interpreted data. MH, AT and JC interpreted the data and revised the manuscript. RH analyzed and interpreted data, and revised the manuscript. SZ conceived and designed the study, analyzed and interpreted data, and revised the manuscript. All authors approved the final version to be published. All authors contributed to the article and approved the submitted version.

## Funding

This work was supported by Novartis Pharmaceuticals Corporation (Investigated-Initiated Trial to SZ), the National Institutes of Health (DK098506 to SZ and Diabetes Research Center P30DK017047), Seattle Institute for Biomedical and Clinical Research and the United States Department of Veterans Affairs. NE is supported by the Dick and Julia McAbee Endowed Postdoctoral Fellowship from the University of Washington, the Francophone Society of Diabetes, the Belgian American Educational Foundation and the Baillet-Latour Found, the Belgian Diabetes Association, the Horlait-Dapsens Foundation, and the Leon Fredericq Foundation. The authors declare that Novartis Pharmaceuticals Corporation was not involved in the study design, collection, analysis, interpretation of data, the writing of this article or the decision to submit it for publication.

## Conflict of Interest

The authors declare that the research was conducted in the absence of any commercial or financial relationships that could be construed as a potential conflict of interest.

## Publisher’s Note

All claims expressed in this article are solely those of the authors and do not necessarily represent those of their affiliated organizations, or those of the publisher, the editors and the reviewers. Any product that may be evaluated in this article, or claim that may be made by its manufacturer, is not guaranteed or endorsed by the publisher.

## Abbreviations

ACE, angiotensin-converting enzyme; ARB, angiotensin II receptor blocker; AT1R, Angiotensin II type 1 receptor; ITT, insulin tolerance test; IVGTT, intravenous glucose tolerance test; GSIS, glucose-stimulated insulin secretion; GLP-1, glucagon-like peptide-1; STZ: streptozotocin; VEH, vehicle.
