# Supplementary material for: Insulinotropic Effects of Neprilysin and/or Angiotensin Receptor Inhibition in Mice
Source: Front Endocrinol (Lausanne). 2022 Jun 6;13:888867. doi: 10.3389/fendo.2022.888867 (PMC9207331; doi:10.3389/fendo.2022.888867)
Supplement: Supplementary file 1 [file DataSheet_1.pdf]

Supplementary Material

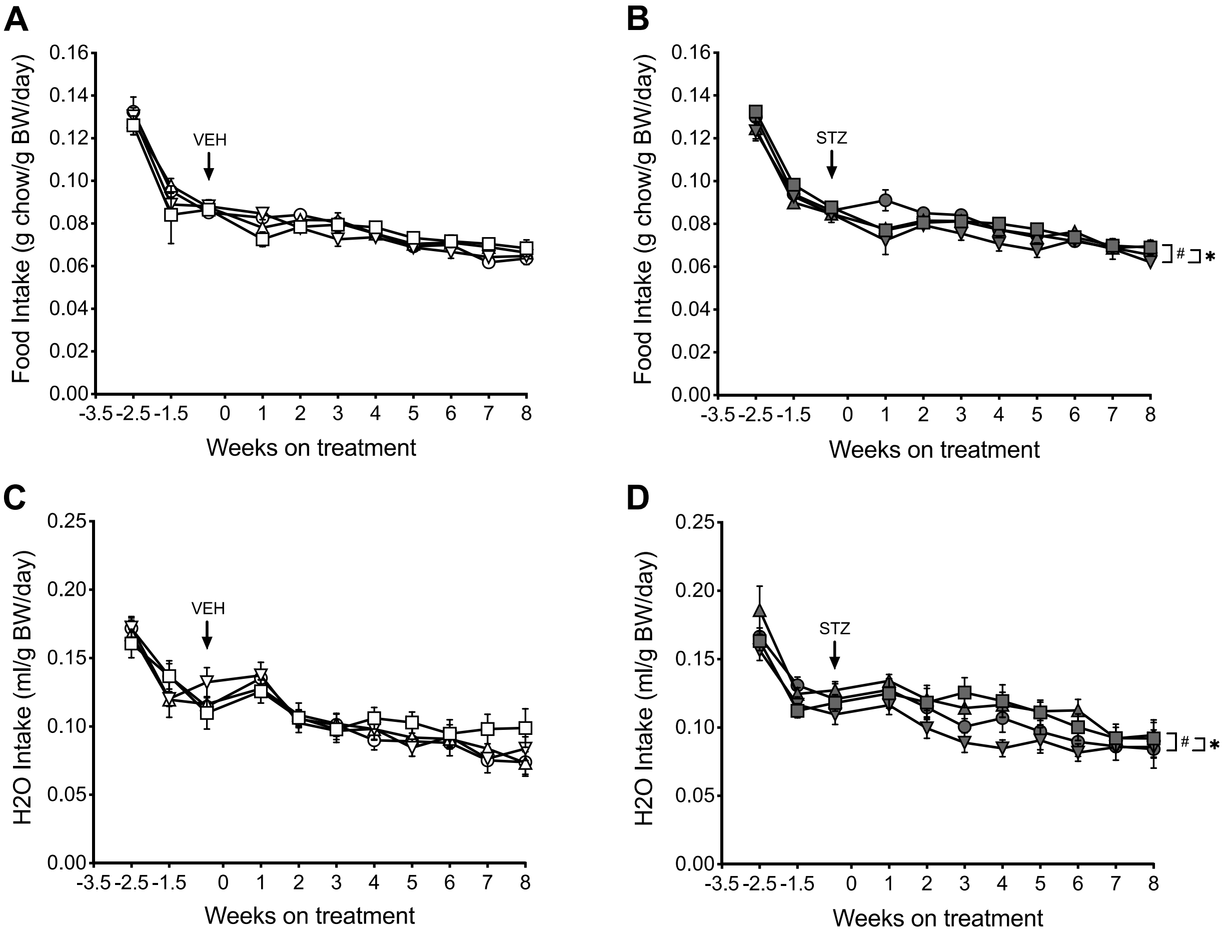

E

	VEH			STZ		
	**SAC**	**VAL**	**S/V**	**SAC**	**VAL**	**S/V**
**Average daily drug intake (mg/kg/day)**	34.6±1.1	39.1±1.3	83.1±3.4	34.4±1.1	39.3±1.1	85.6±2.4
**Average drug dose intake (% of target drug dose)**	72.0±2.3	75.1±2.4	72.9±3.0	71.7±2.4	75.6±2.1	75.0±2.2

Supplementary Figure 1. Food (A, B) and water (C, D) intake over time in high fat-fed mice injected with vehicle (A, C; VEH) or streptozotocin (B, D; STZ) and treated for 8 weeks with control (circles, CTL), sacubitril (inverted triangles, SAC), valsartan (triangles, VAL) or sacubitril/valsartan (squares, S/V). The arrow shows when VEH or STZ was injected. n=7-9 cages/group. *$p \leq 0.05$ SAC vs. CTL, #$p \leq 0.05$ SAC vs. S/V. (E) Average daily drug dose intake during the 8-week treatment period in VEH- and STZ-injected mice treated with sacubitril (SAC), valsartan (VAL) or sacubitril/valsartan (S/V). n=7-9 cages/group.

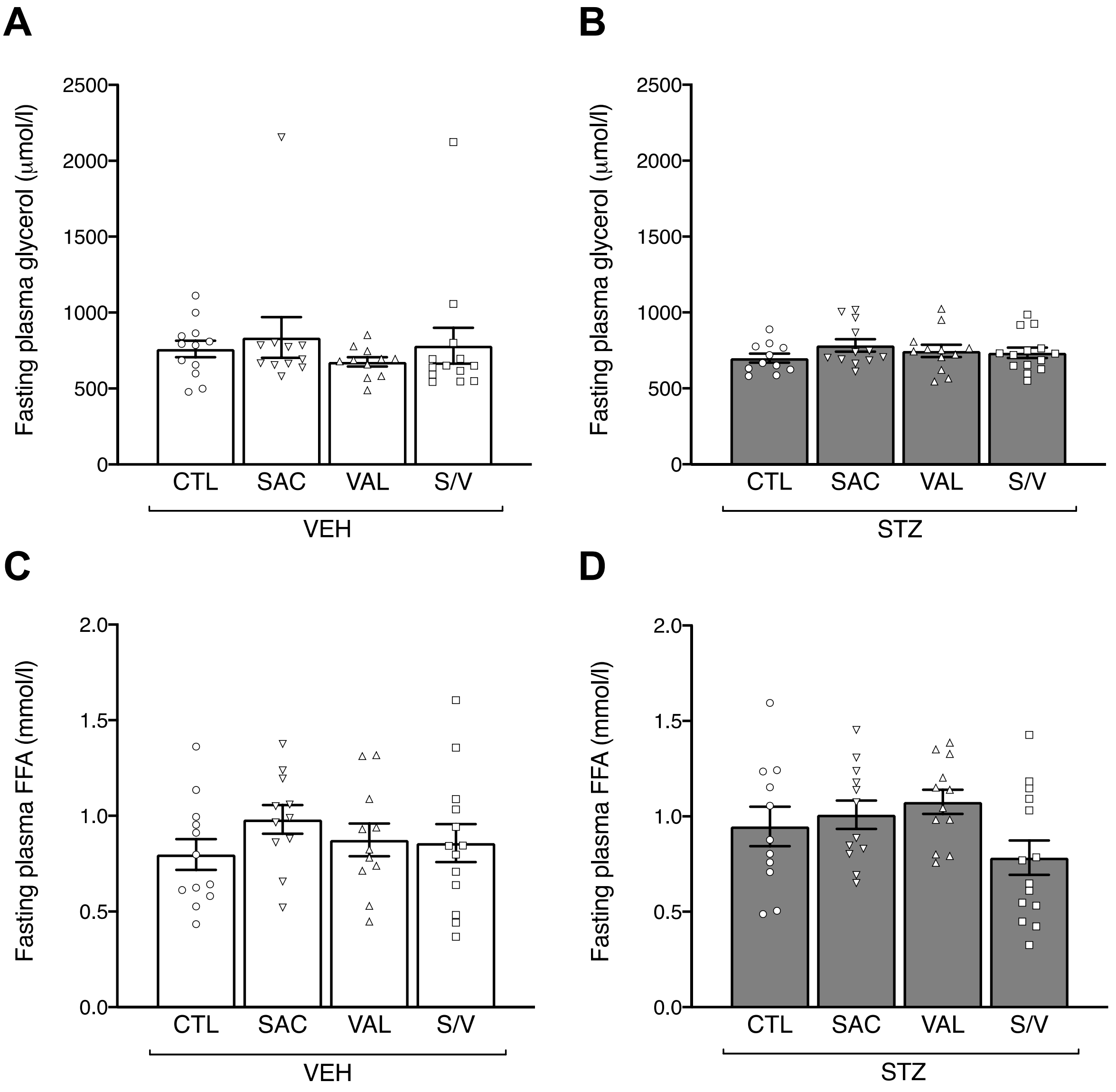

Supplementary Figure 2. Sacubitril, valsartan and sacubitril/valsartan do not alter markers of lipolysis. Fasting plasma glycerol (A, B) and free fatty acids (C, D; FFA) levels at the end of the 8-week treatment period in vehicle (A, C; VEH)- and streptozotocin (B, D; STZ)-injected mice treated with control (CTL), sacubitril (SAC), valsartan (VAL) or sacubitril/valsartan (S/V). n=11-14/group
